# FVRS: a cross-species multi-omics database for fruit and vegetable ripening and senescence

**DOI:** 10.1186/s43897-026-00284-1

**Published:** 2026-10-20

**Authors:** Lili Ma, Yunxiang Wang, Yiting Ren, Caie Wu, Yuanye Jiang, Jiejie Tao, Xuelian He, Xinyi Feng, Tianyu Li, Xu Liu, Jingjing Jin, Jinhua Zuo, Yanyan Zheng

**Affiliations:** 1 Institute of Agri-food Processing and Nutrition, Beijing Academy of Agriculture and Forestry Sciences, Beijing Key Laboratory of Intelligent Processing of Fresh Agricultural Products, Key Laboratory of Vegetable Postharvest Processing, Ministry of Agriculture and Rural Affairs, State Key Laboratory of Vegetable Biobreeding, Beijing Vegetable Research Center, Beijing, 100,097 China; 2 College of Light Industry and Food Engineering, Nanjing Forestry University, Nanjing, Jiangsu, 210,037 China

Fruits and vegetables are dietary staples supplying vitamins, minerals, antioxidants and fibre, and they also provide tractable systems for studying organ development, ripening and senescence. Postharvest ripening and senescence cause softening, colour change, off-flavour and nutrient decline, causing major food loss and economic costs (Giovannoni 2004), so understanding their regulatory networks is a core goal of postharvest biology. While high-throughput sequencing and mass spectrometry have generated extensive multi-omics datasets (Guo et al. 2022; Ma et al. 2024; Tao et al. 2025), these remain scattered across public repositories, supplementary files and species-specific platforms, hampering data reuse, meta-analysis and comparative research. To bridge this gap, we developed FVRS (https://fvripening-senescence.biocloud.net), the first dedicated multi-omics platform for fruit and vegetable ripening and senescence.

The current release of FVRS integrates three core data pillars. The first is a multi-omics repository of 255 datasets from 164 studies, covering 734 samples across 49 species (major fleshy fruits, vegetables and other horticultural crops) and 25 plant families, with 1,378,030 records across five molecular layers—transcriptomics (1,232,390), metabolomics (24,000), proteomics (17,293), epigenomics (91,913) and epitranscriptomics (12,434) (Fig. 1A and B). The second is a reference genome collection of 323 assemblies from 66 species, retained only when both genome FASTA and GFF annotation files were available; 49 species also have multi-omics data, whereas 17 are represented by reference genomes alone (Fig. 1C, Table S1). All genome sequences and annotations are downloadable via the Tools module. The third is a compendium of 298 functionally validated genes reported since 1988 across 186 studies (Table S2), characterized by gain-of-function (transient and stable overexpression) and loss-of-function approaches (RNAi, VIGS, antisense RNA, and CRISPR/Cas9 knockout), and associated with development, ripening, softening, coloration, sugar accumulation, flavour, hormone biosynthesis and signalling, senescence, and postharvest quality (Fig. 1D). Notably, this gene set is subject to literature-driven sampling bias: because of its longer research history, tomato is overrepresented, so the number of validated genes reflects research intensity rather than biological importance; cross-species comparisons based on this set should therefore be interpreted with caution, and conclusions regarding conserved mechanisms should be regarded as preliminary until more data from other species become available.

**Fig. 1:**
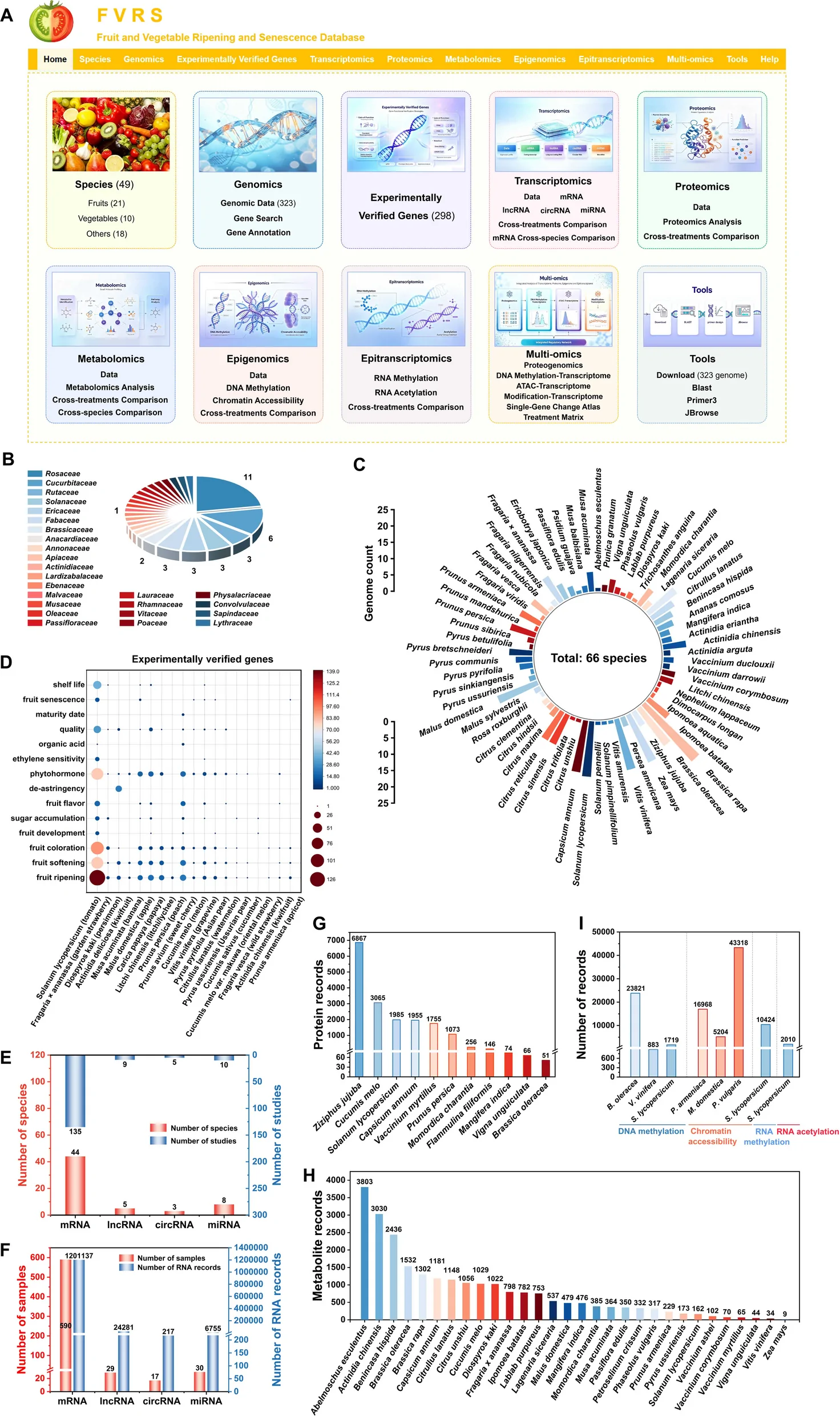
Overview of the FVRS platform architecture and its multi-omics data. **A** The homepage of FVRS, illustrating its 10 main modules and summarizing key database statistics. **B** Pie chart depicting the distribution of species across plant families.** C** Circular bar chart showing genome assembly counts of the 66 included species. **D** Heatmap illustrating the distribution of experimentally verified genes across fruit traits and species. **E** Dual-axis bar chart comparing the numbers of species and studies across transcriptome RNA types. **F** Dual-axis bar chart showing sample counts and RNA record numbers across transcript types. **G** Bar chart of protein record counts across plant species. **H** Bar chart showing the distribution of metabolite records across species. **I** Grouped bar chart of epigenomic and RNA modification records (DNA methylation, chromatin accessibility, RNA methylation and RNA acetylation) across species

Data curation followed predefined quality criteria to ensure reliability and reproducibility. Of the 164 curated studies, 51 were in-house and 113 were published, identified via keyword searches of PubMed, Web of Science and ScienceDirect. Studies were retained only when they provided clearly defined experimental–control or temporal comparisons with at least three biological replicates; those lacking essential metadata were excluded, whereas those missing only secondary fields were kept and annotated as unspecified. The transcriptomics module comprises mRNA-seq data from 590 samples spanning 44 species (135 studies), together with lncRNA, circRNA and miRNA data; the proteomics, metabolomics, epigenomics and epitranscriptomics layers contain 17,293 records from 11 species, 24,000 records from 30 species, 26,423 DNA methylation and 65,490 chromatin accessibility records (from three and three other species, respectively), and 10,424 RNA methylation and 2,010 RNA acetylation records, respectively (Fig. 1E–I). Non-genomic datasets are organized primarily around paired comparisons spanning pre-harvest developmental and ripening stages as well as experimental–control contrasts during postharvest storage. The Transcriptomics, Proteomics, Metabolomics and Epigenomics modules each include a dedicated Data submodule (for Epigenomics, covering chromatin accessibility data only), offering complete quantitative profiles averaged over biological replicates and reported independently of predefined comparisons (Fig. 1A).

FVRS supports gene-level queries by official gene symbols, KEGG- or Swiss-Prot-derived abbreviations, exact gene IDs and accession numbers within the transcriptomics, epigenomics and epitranscriptomics modules. Strict fuzzy matching (e.g., spelling-error tolerance) is not implemented; instead, substring matching is supported—entering "ACO" retrieves ACO1, ACO3 and other genes containing that string—whereas orthology-based cross-species homolog retrieval is not available. Within the Multi-omics module, the Single-Gene Change Atlas enables users to select a species and a gene, then retrieves the molecular alterations of that gene across the transcriptomic, epigenomic and epitranscriptomic datasets, where available. In addition to single-gene queries, two comparative functions are integrated. The Cross-species Comparison function matches mRNA homologs across species via KEGG Orthology (KO), allowing their expression responses to be compared across species under identical or comparable treatments. It returns full differential statistics when experimental conditions are matched, but only regulatory trends otherwise. Cross-species comparison is likewise available for metabolomics, where the same metabolite is matched across species, enabling comparison of metabolite abundances and their associated differential information between species. This function currently covers transcriptomics and metabolomics, but not proteomics, epigenomics or epitranscriptomics. The Cross-treatment Comparison function spans transcriptomics, proteomics, metabolomics, epigenomics and epitranscriptomics, retrieving quantitative values and associated differential information for a queried species and gene/protein/metabolite across multiple treatments, subject to data availability.

Beyond these data resources and analysis tools, FVRS is distinguished by three defining features. First, it applies thematic curation with a treatment-oriented focus: all datasets are manually curated for direct relevance to fruit and vegetable ripening and senescence, with particular attention to physical (cold storage, heat treatment, irradiation, controlled atmosphere), chemical (1-MCP, ethylene, methyl jasmonate, salicylic acid) and biological (antagonistic microorganisms, elicitors) interventions, enabling comprehensive comparative analyses of treatment efficacy and the underlying regulatory mechanisms across diverse horticultural systems. Second, it provides cross-species multi-omic breadth: by consolidating the five omics layers within a single framework, FVRS supports comparative analyses of conserved and species-specific regulatory mechanisms that are not readily achieved by single-species platforms. Third, FVRS enables rapid query of functionally validated regulators: each gene is annotated across 21 fields (genetic background, phenotype, manipulation method and evidence level), and searching "ethylene biosynthesis", for example, retrieves 114 validated regulators with distinct ethylene-related roles, from upstream master regulators (RIN, tomato) to downstream regulators or effectors (MdPG1, apple; MaNAC1/2, banana; PpNAC1/2, peach; CpERF9, papaya).

FVRS is designed to complement, rather than replace, existing community resources, and it overlaps with several established databases while differing in scope and focus. MethBank provides comprehensive, species-wide DNA methylation atlases (Zhang et al. 2023), and m^6^A-Atlas provides species-wide epitranscriptomic atlases, across diverse tissues and developmental contexts (Liang et al. 2024); FVRS shares these molecular layers but restricts its curation to fruit and vegetable ripening and senescence within a treatment-oriented framework, offering complementary depth within a narrower domain. LSD (Leaf Senescence Database) remains a key reference for leaf senescence (Cao et al. 2022), whereas FVRS addresses the distinct biology of postharvest ripening and senescence in edible organs. fruitENCODE assembles epigenomic and transcriptomic data for fleshy fruits (Lü et al. 2018), and TomOmics provides deep multi-omics integration for tomato (Wang et al. 2026); FVRS overlaps with these in its multi-omics coverage but extends the framework to 49 horticultural species and five omics layers within a single cross-species platform. Although all of these platforms offer browsable, downloadable multi-omics data and gene-level queries, each retains unique strengths within its own domain; FVRS differs primarily in its treatment-oriented, crop-specific curation and cross-species comparison, and is positioned as a thematic, crop-specific complement to, rather than a substitute for, these established resources.

Beyond data compilation, FVRS offers interactive querying, visualization, and downloadable results, complemented by integrated tools for sequence alignment (BLAST), primer design (Primer3), and genome visualization (JBrowse). With twice-yearly updates incorporating new multi-omics datasets and expanded species coverage, FVRS is expected to advance postharvest biology research by enabling identification of conserved regulatory mechanisms, development of precision preservation technologies, and molecular breeding for improved shelf life and quality.

## Supplementary Information

Supplementary Material 1.

Supplementary Material 2.

Supplementary Material 3.

Supplementary Material 4.

## Data Availability

The data integrated into FVRS were extracted from the corresponding publications and their supplementary files, and are accessible at https://fvripening-senescence.biocloud.net. For traceability, FVRS provides accession numbers linking to public repositories (e.g., NCBI SRA, GEO, PRIDE) wherever available, while every original study is accompanied by its DOI for access to the corresponding article. A table of all accession numbers is additionally provided on the download page under the "Tools" section of the FVRS website. Additional materials supporting this study are available from the corresponding author upon reasonable request.
