# Supplementary material for: FVRS: a cross-species multi-omics database for fruit and vegetable ripening and senescence
Source: Mol Hortic. 2026 Oct 20;6:79. doi: 10.1186/s43897-026-00284-1 (PMC13681938; doi:10.1186/s43897-026-00284-1)
Supplement: Supplementary file 1 [file 43897_2026_284_MOESM1_ESM.docx]

**Database sustainability**

The FVRS database is maintained as a core resource of the Institute of Agri-food Processing and Nutrition, Beijing Academy of Agriculture and Forestry Sciences (BAAFS). Its long-term sustainability is ensured through scheduled updates, standardized data quality control, a modular technical infrastructure, a dedicated institutional team, contractual maintenance arrangements, secured funding, and a formal commitment to free and open access.

**Scheduled updates**

FVRS will be updated twice a year, in June and December. All changes are thoroughly documented in a publicly accessible changelog, allowing users to track additions, corrections, and functional updates over time.

**Data quality control**

Each update is processed through a standardized curation pipeline that integrates automated validation, manual expert review, and cross-referencing against authoritative primary databases (e.g., NCBI and UniProt). This pipeline ensures the reliability and consistency of the curated data.

**Technical infrastructure**

The database employs a modular architecture that supports independent updates to individual modules. This minimizes system downtime and enables continuous, incremental enhancement of the database.

**Institutional and personnel responsibilities**

FVRS operation and maintenance involve a clear division of responsibilities between BAAFS (the data owner and lead institution) and Beijing Biomarker Technologies Co., Ltd. (the contracted technical service provider), as formalized in a maintenance-service agreement.

Institutional responsibilities:

a. BAAFS (Party A) serves as the data owner and lead institution. It is responsible for providing raw data, defining functional requirements, issuing content-update instructions, paying maintenance fees, and retaining full ownership of all database data.
b. Beijing Biomarker Technologies Co., Ltd. (Party B) serves as the contracted technical service provider. It is responsible for daily system maintenance, bug fixes, webpage and content updates, server-environment configuration, and SSL certificate support. Party B is contractually obligated to guarantee free public access during the service term, to provide continuous operation and maintenance throughout the service term (through July 31, 2037), to perform weekly full data backups, and to implement safeguards against data leakage, tampering, and loss.
c. Intellectual property and data security: The institute retains full data ownership, and the service provider may use the data only within the authorized scope. Mutual confidentiality obligations extend for ten years after termination. In the event of data leakage or loss, the service provider is liable for compensation and data-recovery assistance.

Personnel responsibilities:

A dedicated core team within the institute—comprising two bioinformaticians and one system administrator, led by Dr. Yanyan Zheng, a co-author of this manuscript—oversees daily operations, server maintenance, curation, and future data integration. Routine operations are coordinated through designated project contacts to ensure traceable accountability:

a. Institute contact and team lead: Dr. Yanyan Zheng — leads the core maintenance team, submits maintenance requests, issues update instructions, and receives deliverables from the service provider.
b. Service provider contact: Ms. Yanyan Guo — executes maintenance tasks, responds to issues, and reports operational status to the institute.

All maintenance requests, issue reports, and change notifications are communicated through these designated contacts, ensuring clear and traceable accountability throughout the operation workflow.

**Funding**

The maintenance-service agreement was signed in June 2026. Following an initial system deployment period, the database entered the maintenance phase in July 2026. The maintenance service is provided free of charge for the first service year (July 2026 – July 2027). From the second year through the end of the service term on July 31, 2037, BAAFS allocates a dedicated annual budget for database maintenance (Section 2 of the Agreement).

This support is complemented by research grants from multiple sources, including the Natural Science Foundation of Beijing (6242012, 6254030), the National Natural Science Foundation of China (32472800), the Beijing Rural Revitalization Agricultural Science and Technology Project (NY2602550000), the Special Innovation Ability Construction Fund of the Beijing Academy of Agriculture and Forestry Sciences (KJCX20261805, KJCX20240405), and the Beijing Academy of Agriculture and Forestry Sciences Foundation for Excellent Young Scientists (YKPY202614). The institute is committed to pursuing additional collaborations and institutional support to sustain and expand the database beyond the current funding period.

**Data security and backup**

In accordance with the contract, the service provider performs a full backup once a week and retains it properly during the term of the agreement, and implements safeguards against unauthorized access, tampering, and data loss (Sections 3.1 and 4 of the Agreement). All data in the database are owned by the institute. Upon termination of the agreement, the service provider shall delete or transfer the relevant data as required by the institute, ensuring that the institute retains full control of the data and can migrate them to an alternative system if necessary.

**Accessibility assurance**

FVRS will remain freely and openly accessible to the entire research community, with no registration or subscription barriers. This commitment is formally reinforced by the maintenance-service agreement: Section 3.6 mandates that, at the institute's request, the website shall remain freely and publicly accessible throughout the entire maintenance period, and the service provider shall not impose any access restrictions or charges. The service term extends to July 31, 2037 (Section 1), ensuring that free and unrestricted access is contractually guaranteed through July 31, 2037. The institute is committed to providing long-term, uninterrupted service to support the broader scientific community.
