# Supplementary material for: FVRS: a cross-species multi-omics database for fruit and vegetable ripening and senescence
Source: Mol Hortic. 2026 Oct 20;6:79. doi: 10.1186/s43897-026-00284-1 (PMC13681938; doi:10.1186/s43897-026-00284-1)
Supplement: Supplementary file 2 [file 43897_2026_284_MOESM2_ESM.docx]

**Data curation procedures**

1. **Data processing**

This section describes how quantitative values are handled in the FVRS database.

- **Data source:** Quantitative values are extracted directly from the original publications or their supplementary files.
- **Averaging across biological replicates:** For each condition, the expression or abundance values of the biological replicates are averaged, and a single representative value per condition is stored in the database. Individual replicate-level values are not provided, because all datasets are uniformly represented as averaged values to ensure comparability across studies.
- **No further normalization or imputation:** No additional normalization or imputation is applied to the quantitative values, and missing values are not inferred or imputed.
- **Scope:** This processing applies to all datasets in the transcriptomics, proteomics, and metabolomics modules, as well as to chromatin accessibility data in the epigenomics module. DNA methylation and epitranscriptomics data are not provided as complete quantitative matrices, because the original publications generally report modification sites or peaks rather than sample-level quantitative levels.
- **Traceability:** Each dataset is linked to its original publication and, where available, to the reported repository accession number, so that users can trace every value back to its source.

2. **Metadata quality control**

FVRS applies a tiered rule to handle incomplete metadata:

- **Excluded:** Datasets are excluded when the missing metadata prevent quantitative data from being reliably interpreted or compared (e.g., unknown species/cultivar, undefined treatment conditions, ambiguous sample grouping, or completely missing time information, i.e., neither pre-harvest developmental time nor post-harvest storage duration is available).
- **Retained and annotated:** Datasets are retained when only secondary fields are missing (e.g., exact storage temperature, specific harvest maturity indicators, or stage-based or relative descriptions without exact values, such as "harvested at the green-mature stage" without days after flowering, or "stored at room temperature" without a specific temperature value); the missing fields are labeled as "-" (i.e., unspecified).
- **No imputation:** No imputation or inference is performed for missing metadata.

**Relationship between sampling time and storage duration:** In this database, "sampling time" refers to the pre-harvest developmental time point (e.g., 20 days after flowering), whereas post-harvest sampling points are recorded in the "storage duration" field. Therefore, for a sample taken after 15 days of storage, the storage duration is recorded as 15 days, and the pre-harvest sampling-time field is left empty (indicating that the study did not report a pre-harvest time, or that the sampling point belongs to the post-harvest period). In such cases, an empty pre-harvest sampling-time field is not considered missing data.
